# Detection of the ORF1 Gene Is an Indicator of the Possible Isolation of Severe Acute Respiratory Syndrome Coronavirus 2

**DOI:** 10.3390/pathogens11030302

**Published:** 2022-02-27

**Authors:** Kazuya Shirato, Masatoshi Kakizaki, Yuriko Tomita, Miyuki Kawase, Makoto Takeda

**Affiliations:** 1 Department of Virology III, National Institute of Infectious Disease, 4-7-1 Gakuen, Musashimurayama 208-0011, Tokyo, Japan; kakizaki@niid.go.jp (M.K.); kawase@nih.go.jp (M.K.); mtakeda@nih.go.jp (M.T.); 2 Influenza and Respiratory Virus Research Center, National Institute of Infectious Disease, 4-7-1 Gakuen, Musashimurayama 208-0011, Tokyo, Japan; ymtomita@niid.go.jp

**Keywords:** coronavirus diseases 19 (COVID-19), severe acute respiratory syndrome coronavirus 2 (SARS-CoV-2), real-time RT-PCR, virus isolation, ORF1a

## Abstract

In the ongoing coronavirus diseases 2019 (COVID-19) pandemic, caused by severe acute respiratory syndrome coronavirus 2 (SARS-CoV-2), real-time RT-PCR based diagnostic assays have been used for the detection of infection, but the positive signal of real-time RT-PCR does not necessarily indicate the infectivity of the patient. Due to the unique replication system of the coronavirus, primer/probe sets targeted nucleocapsid (N) and spike (S) protein detect the abundantly synthesized subgenomic RNAs as well as the virus genome, possibly making the assay unsuitable for estimation of the infectivity of the specimen, although it has an advantage for the diagnostic tests. In this study, the primer/probe set targeting the open reading frame 1a (ORF1a) gene was developed to specifically detect viral genomic RNA. Then the relation between the ORF1a signal and infectivity of the clinical specimens was validated by virus isolation using VeroE6 cells, which constitutively express transmembrane protease, serine 2, (VeroE6/TMPRSS2). The analytical sensitivity of developed ORF1a set was similar to that of previously developed N and S sets. Nevertheless, in the assay of the clinical specimen, detection rate of the ORF1a gene was lower than that of the N and S genes. These data indicated that clinical specimens contain a significant amount of subgenomic RNAs. However, as expected, the isolation-succeeded specimen always showed an RT-PCR-positive signal for the ORF1a gene, suggesting ORF1a detection in combination with N and S sets could be a more rational indicator for the possible infectivity of the clinical specimens.

## 1. Introduction

The worldwide outbreak of coronavirus disease 2019 (COVID-19), caused by severe acute respiratory syndrome coronavirus 2 (SARS-CoV-2), originated in Wuhan, China, in December 2019 [1]. As of 29 September 2021, a total of 232,636,622 confirmed cases and 4,762,089 deaths have been reported globally [2].

COVID-19 is usually diagnosed based on the detection of the SARS-CoV-2 genome using genetic diagnostic methods, such as real-time RT-PCR assays. This is a highly sensitive and specific method to detect the genomes of infectious agents because the presence of several copies of the targeted region’s sequence are enough to obtain a positive result. Early in the outbreak of SARS-CoV-2, it was reported that three primer/probe sets (targeted open reading frame (ORF1), envelope (E), and nucleocapsid (N) genes) developed for the detection of the SARS-like bat beta-coronavirus could be used in the detection of SARS-CoV-2 [3]. These were adopted for testing by the World Health Organization (WHO) [4,5]. In Japan, a primer/probe set for the detection of SARS-CoV-2 that targeted the N protein region was developed by our laboratory (National Institute of Infectious Diseases, NIID) in January 2020. This NIID-N2 set was used for national surveillance. Soon after, another primer/probe set to detect the spike (S) protein region (NIID-S2 set) was also developed and used for national surveillance together with NIID-N2 set [6,7]. In the very early stages of the pandemic, the Centers for Disease Control and Prevention in the United States (US-CDC) also developed real-time RT-PCR-based assays targeting the N protein region [8]. 

Given the unique replication system of the coronavirus, two strategies have been proposed. One is that the full genome-size negative-strand RNA is transcribed from the genomic positive-strand RNA, and acts as a template for producing subgenomic mRNAs (sgmRNAs) [9]. The other is that the negative-strand counterpart for the genome and for each sgmRNA is synthesized from the genomic positive-strand RNA [10]. In either case, different lengths of sgmRNAs are synthesized as many as the number of coding proteins because only the protein encoded endmost of 5′-side is translated in conventional theory [11]. Recent studies suggest that various length of non-canonical sgmRNAs are synthesized during virus replication besides canonical sgmRNAs, which account for more than 90% of the total sgmRNAs [12,13]. Thus, most of the canonical and non-canonical sgmRNAs contain N protein sequence at the 3′-end, i.e., the most N protein sequences are produced in the coronavirus-infected cells [13,14]. The N protein region is therefore the most common target for the detection of coronaviruses, and many N-targeted primer/probe sets for the detection of SARS-CoV-2 have been developed at the beginning of the pandemic as mentioned above.

During the COVID-19 pandemic, real-time RT-PCR-based diagnostic assays have been used for the detection of SARS-CoV-2 infections; however, correlation between the positive signal of real-time RT-PCR and the possibility of virus spread from patient became a problem since PCR-negative results had been required for discharge [15,16,17]. First, real-time RT-PCR assays can detect viral RNA but not infectious viruses. Second, as described above, the primer/probe set is targeted for the N protein region likely to detect not only genomic RNA, but also abundantly synthesized sgmRNAs, indicating the positive signal by real-time RT-PCR with the N-targeted set traces the history of viral replication, because it may detect residual sgmRNAs even in fully recovered patients. Some reports described that detection of sgmRNA was not an indicator of active replication [18]; other reported sgmRNAs were useful to identify active virus replication [19,20,21]. Nevertheless, a direct link between PCR results and infectivity has been demanded. 

In this study, to support the understanding of the infectivity of PCR-positive specimen, a primer/probe set targeted ORF1a region with equivalent analytical sensitivity with the NIID-N2 set was developed to increase the possibility of genome RNA detection, and the relation between its positive signal and virus isolation was analyzed. Because the ORF1a region sequences were not contained in canonical sgmRNAs, which accounted for majority of sgmRNA and the DNA, nanoball and nanopore sequencing data showed low coverage for the ORF1a region [13]. 

## 2. Results

### 2.1. Development of the ORF1a Set for SARS-CoV-2 Detection

**Figure 1:**
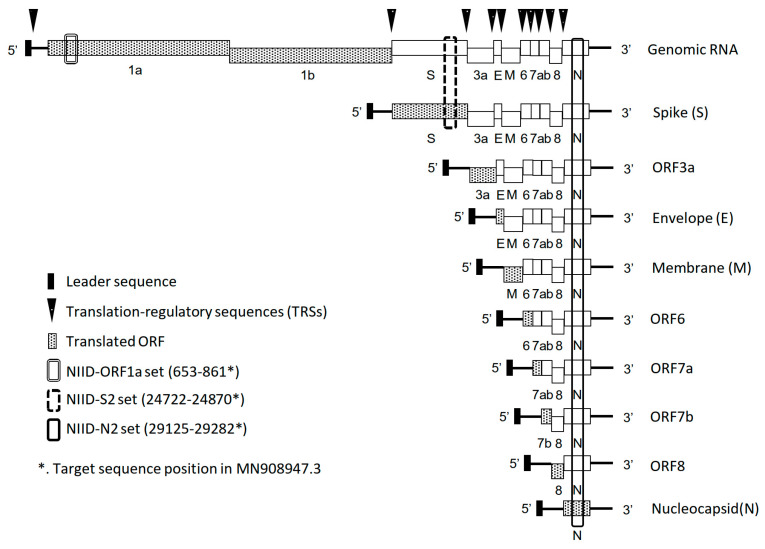
Schematic image of coronavirus canonical subgenomic mRNAs and the position of the region targeted by real-time RT-PCR. Black bar, leader sequence; black inverted triangle, translation-regulatory sequence; gray hatching, translated ORF; double lined box, NIID-ORF1a; break lined box, NIID-S2 set; black box, NIID-N2 set.

Coronaviruses produce sgmRNAs during their replication, and nine to ten major sgmRNAs have been identified for SARS-CoV-2 [12,13]. As shown in Figure 1, genomic RNA and canonic sgmRNAs contain the NIID-N2 targeted region, and the NIID-S2 targeted region is contained to within both the genomic RNA and the sgmRNA encoding the S protein. By contrast, the ORF1 region sequence is not in canonic sgmRNAs. Although some non-canonic sgmRNA contains ORF1a region, the amount of non-canonic sgmRNA is quite low [13]. Therefore, to detect this genomic RNA with high probability, a primer/probe set targeting the ORF1a gene was developed and validated. First, the analytical sensitivity was confirmed using synthesized RNA (Table 1). The analytical sensitivity of NIID-ORF1a was 2.5 copies, which was close to those of NIID-N2 (1.4) and NIIDS2 (1.4). Although the binding affinities of the primer/probe sets might be different, the analytical sensitivities of these three sets were similar. The NIID-ORF1a set did not react nonspecifically with other respiratory viruses (Supplemental Table S1), indicating that it could be equivalently used with previously reported sets for the detection of SARS-CoV-2 RNA [6,7]. 

### 2.2. Difference in Detection Efficiency of Viral RNAs in Clinical Specimens and Virus Culture Supernatants (Virus Stocks) by Primer/Probe Set

Then, the performance of the NIID-ORF1a set was evaluated using clinical specimens obtained during national surveillance in Japan. Among 25 nasopharyngeal swabs, 15 were negative and 10 were positive for SARS-CoV-2 by NIID-N2 and NIID-S2 sets (Supplemental Table S2). All negative specimens were identified as negative by the NIID-ORF1a set; however, two specimens with a high crossing point (Cp) value determined to be positive by the NIID-N2 and the NIID-S2 sets were negative when tested with the NIID-ORF1a set. The Cp value by NIID-ORF1a was always higher than those by NIID-N2 and NIID-S2. Similarly, the Cp value by NIID-S2 was always higher than those by NIID-N2. These different sensitivities were caused by several reasons and one of them might be a significant amount of sgmRNAs in specimens. A similar analysis was performed using a culture supernatant (a virus stock) of VeroE6/TMPRSS2 cells infected with SARS-CoV-2 WK-521 isolate. Viral RNAs were purified from the virus stock, and the copy number of viral RNAs was calculated by the NIID-N2 set. When the analytical sensitivities were estimated using the viral RNAs from the virus stock, NIID-ORF1a set showed lower analytical sensitivity (14.1) than NIID-N2 (6.5) andNIID-S2 (4.4) sets in different from using control RNA (Table 1). These results appeared as if the analytical sensitivity of NIID-ORF1a set was lower than those of NIID-N2 and NIID-S2 sets. However, the cause of these results was presumably due to the replication system of coronavirus as previously described, i.e., the difference in the amount of viral RNA for each target sequence in different primer/probe sets. We speculated that the genomic RNA with the ORF1a sequence, but not sgmRNAs and miscellaneous RNAs, is encapsidated and protected in the enveloped virion. Therefore, to exclude sgmRNAs and miscellaneous RNAs, the virus stock was treated with RNase A and then viral RNAs were extracted, then the copy number of viral RNAs was calculated by the NIID-N2 set. This treatment improved the analytical sensitivity of NIID-ORF1a set from 14.1 to 4.4 (Table 1), indicating that the viral RNA copy number determined by a primer/probe set for N gene deviates from the genomic RNA copy number under the conditions generally used in real-time RT-PCR tests for SARS-CoV-2 detection.

### 2.3. Analytical Sensitivity of Viral Isolation with VeroE6/TMPRSS2

To estimate the infectivity of a specimen, virus isolation can provide a helpful clue. We previously reported that serine protease, TMPRSS2-expressing VeroE6 (VeroE6/TMPRSS2) cells are suitable for the isolation of SARS-CoV-2 [22]. The relation between the real-time RT-PCR results and virus isolation have been described in several studies [23,24,25]. However, a reliance on the cycle threshold (Ct) value can be misleading, because it depends on the chosen primer/probe set, reagent, and the analyzed condition (threshold and baseline). In addition, the value calculated based on the intersection of the amplification curve and the threshold value differs from the Cp calculated using the 2nd derivative maximum method. For these reasons, the viral RNA copy number should be used for the evaluation. To accurately determine the isolation efficiency, the analytical sensitivity of VeroE6/TMPRSS2 cells in the isolation of SARS-CoV-2 was evaluated (Table 2). Six different isolates were propagated using VeroE6/TMPRSS2 and the supernatants were treated with RNase A. After RNA extraction, the copy numbers were determined by the NIID-N2 set. Based on the copy numbers, virus stocks were serially diluted in virus-transport medium-containing nasopharyngeal swab, and then the dilutions were inoculated onto VeroE6 or VeroE6/TMPRSS2 cells. The Wuhan type of virus (WK-521 strain) did not differ in isolation efficiency, while those of recent variant of concerns (VOCs) were different with or without TMPRSS2. The averages of analytical sensitivity (the minimum viral RNA copy number required for virus isolation) in VeroE6 and VeroE6/TMPRSS2 were log 2.8 (681.3) and log 1.6 (43.0) copies, respectively, indicating better isolation efficiency of SARS-CoV-2 in VeroE6/TMPRSS2 than in the non-protease producing VeroE6 cells. 

### 2.4. Virus Isolation with Low Copy Number Specimens

For negative infectiousness confirmation such as in determination of patient discharge, determining the infectivity of the specimen is often challenging. In a previous study [24], a Ct of 28.1 by the NIID-N2 set was the cut-off value and resulted in 87.5% successful viral isolation. In our real-time RT-PCR system, a Cp of 28 corresponds to log 3.5 copies per reaction (contains 11.7 μL of specimen). In this study, specimens with a Cp > 28 (28.51–38.67) by NIID-N2 set (54 samples) or negative by NIID-N2 but positive (Cp 34.86–37.66) by NIID-S2 (three samples) were used in virus isolation (Table 3). As described above, about 50 copies of virus RNAs resulted in 50% successful isolation; therefore, the specimen, which contained at least 100 copies of viral RNAs (determined by NIID-N2), was inoculated onto the cells. The six specimens were positive for virus isolation (success rate of 10.5%) and all of these isolation-positive specimens were PCR positive by the NIID-ORF1a set. The average of rate of log copy numbers were 1.11 ± 0.16 (NIID-N2/NIID-ORF1a) and 1.46 ± 0.18 (NIID-S2/NIID-ORF1a) and these were statistically significant (*p* = 0.001 and *p* < 0.001), indicating the amount of NIID-N2 and NIID-S2 targeted sequences were larger than that of NIID-ORF1a, as described above. In addition, among the 57 specimens, the number of positives were 54, 52, and 44 for NIID-N2, NIID-S2, and NIID-ORF1a, showing a significantly lower positive in NIID-ORF1a (*p* = 0.01). This supports the hypothesis that N- and S-targeted sets tend to detect abundant sgmRNAs. The range of copy numbers (determined by NIID-N2) for successful isolation was 100 to 800 (average 224.5 copies). The lowest the copy number among the virus-isolated specimen determined using the NIID-ORF1a set was log 1.21 copies per reaction (log 3.14 copies/mL). Not all NIID-ORF1a positive specimens could be isolated, but infectious virus was never isolated from NIID-ORF1a negative specimen. Thus, in combination with N- and S-targeted sets, the real-time RT-PCR positive by ORF1a-targeted set, which likely detects the virus genome, could be a rational indicator for the successful virus isolation from specimens, which showed high Ct or Cp values in the diagnostic real-time RT-PCR test, and possible infectivity of the patients. 

### 2.5. Primer/Probe Mismatches in NIID Assays for VOCs

In a previous study, we evaluated the performances of NIID-N2 and NIID-S2 assays for VOCs up to Delta [7,26]. Variations of mismatches in primer/probe region were seen and some of them did not affect the analytical sensitivities. The mismatches located near 3′-end of primers affected the analytical sensitivities; however, the frequencies of mismatches were lower in VOCs than those in Wuhan type [7,26]. Currently, the Omicron variant has infested the world; therefore, the primer/probe mismatches of NIID-assays were evaluated using the genomic sequences of Omicron variant deposited in the GISAID database (Global Initiative on Sharing Avian Influenza Data, https://www.gisaid.org, accessed on 27 Febuary 2022) in the first half of February 2022 as previously described [7,26]. About 290,000 to 350,000 sequences were available for the calculation and the occurrence of mismatches were low (0.1% to 0.3%) except the probe of the NIID-S2 set and forward primer of the NIID-ORF1a set (supplemental Table S3). In the probe of the NIID-S2 set, T to G substitution on 11th nucleotide (NIID-S2_P_T11G) accounted for 1.54% of available sequences. The analytical sensitivity of the NIID-S2 set for the original sequence was 1.4 (Table 1) and that for this mismatched sequence was 2.5. Although the analytical sensitivity for NIID-S2_P_T11G was statistically lower that for original sequence (*p* = 0.01, *n* = 4), the NIID-S2 set could detect 2.5 copies of viral RNA, suggesting the set showed sufficient performance (less than five copies) as a diagnostic assay. In the forward primer of the NIID-ORF1a set, T to G substitution of 18th nucleotide accounted for 11.8% of available sequences. The analytical sensitivity of NIID-ORF1a set was 2.5 (Table 1) and that for the mismatched sequence was 5.6, suggesting this mismatch also did not affect the analytical sensitivity, though it located near the 3′ end (*p* = 0.129, *n* = 3). These suggest that the NIID assays still work for the detection of VOCs including Omicron variants.

## 3. Discussion 

The purpose of this study was developing an ORF1a-targeted primer/probe set to detect SARS-CoV-2 RNA and evaluate its relation to possible virus isolation. This study showed the NIID-ORF1a set could be helpful to presume the presence of infectious virus in the specimen by the real-time RT-PCR results in combination with NIID-N2 and NIID-S2 sets. 

Due to the unique replication system of the coronavirus, the region to be targeted by real-time RT-PCR must be carefully selected. Because the N gene is the most abundant in coronavirus-infected cells [27,28], it was the first target in the construction of a primer/probe set. However, the detection of abundantly synthesized sgmRNAs does not always lead to the presence of an infectious virion. After the exposure, the virus enters an incubation period, replication period, and declining period with the counter immune-system. [29]. Viral genes can be detected by genetic diagnostic testing during both the active replication and tapering periods but a one-time cannot distinguish between the two. Therefore, primer/probe sets, which can detect both the genome and sgmRNAs, such as the targeted N gene, are useful in a screening test to identify possibly infected individuals. In contrast, primer/probe sets, which detect only the genome RNA, are not suitable for screening tests due to their lower sensitivity in practical use than sgmRNA-detectable primer/probe sets. This study showed that virus isolative specimens showed PCR positive for the ORF1a gene as well as N and S, but not vice versa. This suggest that the ORF1a primer/probe set is more useful than N or S primer/probe sets for testing the possible presence of infectious virus in the specimen. Thus, different target primer/probe sets should be used depending on the purpose of detection of the coronaviruses, i.e., N and S sets are for the diagnosis and ORF1a set for the prediction of the presence of infectious virus. 

We also note that it is important to understand the difference between Ct and Cp values and understand that it is meaningless to compare Ct values directly in tests conducted using different primer/probe sets, analysis conditions, reagents, instruments, and so forth. This was demonstrated in our study, in which there were two to three differences between the NIID-N2, NIID-S, and NIID-ORF1a sets, but the specimens contained similar virus copy numbers. The online publication from Public Health of England provides a useful explanation of the Ct value [29]. 

This study also suggested that VeroE6/TMPRSS2 cells might be more highly sensitive then VeroE6 cells for the isolation of VOCs of SARS-CoV-2, especially the current Delta-related variants. Although the isolation and maintenance of SARS-CoV-2 using VeroE6 cells without protease induce a deficiency of the furin-recognition site [30,31], which is the S1/S2 cleavage site, this is not the case in TMPRSS2-expressing cells [32]. In previous studies, we showed that clinical isolates of coronaviruses prefer the early-endosomal pathway using TMPRSS2 for cell entry to the late-endosomal pathway using cathepsins [33,34]. Therefore, VeroE6/TMPRSS2 cells are well-suited for the isolation and maintenance of clinical SARS-CoV-2 viruses. Our study also showed that about 50 copies of virus is sufficient for 50% isolation. Accordingly, in virus isolation, an amount of specimen, large enough to contain >50 copies of SARS-CoV-2 RNA, calculated based on a RT-PCR test, could improve the rate of successful virus isolation.

The analysis of primer/probe mismatches of the NIID assays used in this study suggested that the NIID assays still can work for the detection of Omicron variant, which was dominant in current epidemiology of SARS-CoV-2 [35], though some distinctive mismatches were seen. The consistency of primer/probe sequences are important to guarantee the performance of the real-time RT-PCR-based assay. Therefore, it needs to continue the monitoring of the trend of circulating variants and their sequences. 

In summary, the study suggested that the combination of SARS-CoV-2 detection using the ORF1a primer/probe set, as well as N- and S-targeted sets, and viral isolation using VeroE6/TMPRSS2 cells can facilitate the detection of infectious virus in a specimen. In this study, our NIID assays were used for the evaluation; however, other ORF1a-targeting assays could also be used for the same purpose. 

## 4. Materials and Methods

### 4.1. Cells and Viruses

VeroE6 cells (American tissue culture collection (ATCC), CRL-1586) and VeroE6 cells that constitutively express transmembrane protease, serine 2 (TMPRSS2), (VeroE6/TMPRSS2), [22,36] were used in this study. The cells were maintained in DMEM (D5796) (Sigma-Aldrich, St. Louis, MO, USA) containing 10% fetal bovine serum (FBS) and antibiotics. The following SARS-CoV-2 isolates, obtained using VeroE6/TMPRSS2 cells, were used: AI/I-004/2020, GISAID. EPI_ISL_407084 (Wuhan), TY/WK-521/2020 (Wuhan), EPI_ISL_408667; QH-328-073/2020, unregistered (D614G); QHN001/2020 (Alpha), EPI_ISL_804007; TY8-612 (Beta), EPI_ISL_1123289; TY11-330-P1 (Delta), EPI_ISL_2158613; TY11-927-P1 (Delta), EPI_ISL_2158617. Other respiratory viruses described in previous studies [7,37] were used for the validation of the primer/probe set. In addition, human coronaviruses (HCoV) isolates obtained using human bronchial tracheal epithelial cells in air-liquid interface culture [34] (229E, Fukushima/H829/2020 and Fukushima/H832/2020; NL63, Fukushima/H219/2018; OC43, Fukushima/O120/2017, Fukushima/H148/2018, Fukushima/H189/2018, Fukushima/H478/2019, and Fukushima/H509/2019; HKU1, Fukushima/H815/2020, Fukushima/H821/2020, and Fukushima/O943/2020) were used. SARS-CoV-2 isolates were titrated using VeroE6/TMPRSS2 cells as previously described [22]. 

### 4.2. Specimens

Pharyngeal swabs, nasal swabs, and sputum collected in virus-transport medium were obtained during national surveillance tests from February to May in 2020, and stored at −80 °C, at almost same time, indicating the influence by the storing condition was able to be considered almost the same for all. Additional nasopharyngeal and nasal swabs were obtained from Discovery Life Sciences (Los Osos, CA, USA). All swabs were used with the approval of the Research and Ethical Committee for the Use of Human Subjects of the National Institute of Infectious Diseases, Japan (approval #1001 and 1091). Nucleic acid was extracted using TRIzol LS, TRIzol reagent (Thermo Fisher Scientific, Waltham, MA, USA), QIAamp Viral RNA Mini Kits (Qiagen, Hilden, Germany), and/or the QIAamp 96 Virus QIAcube HT kit (Qiagen) in accordance with the manufacturers’ instructions.

### 4.3. Real-Time RT-PCR for the Detection of SARS-CoV-2

The NIID-N2 and NIID-S2 assays were conducted as previously described [6,7]. Primers and the probe targeting ORF1a were designed using Primer 3 software (ver. 4.0, http://bioinfo.ut.ee/primer3-0.4.0/, accessed on 27 Febuary 2022) based on the sequence of a Japanese viral isolate (Japan/TY/WK-521/2020, EPI_ISL_408667) as follows: SARS-CoV-2-NIID-ORF1a-F1, 5′-GGAGCTGGTGGCCATAGTTA-3′; SARS-CoV-2-NIID-ORF1a-R2, 5′-TCAAGAGGGTAGCCATCAGG-3′; SARS-CoV-2-NIID-ORF1a-P1, FAM-5′-GGCGACGAGCTTGGCACTGA-3′-BHQ1. The concentration of the primers and probe for the ORF1a set were as follows: NIID-ORF1a-F1, 500 nM; NIID-ORF1a-R2, 500 nM; NIID-ORF1a-P1, 200 nM. Real-time RT-PCR was performed using QuantiTect Probe RT-PCR kits (Qiagen) and LightCycler systems (480, 96, and nano; Roche, Basel, Switzerland), which equipped the 2nd derivative maximum method for data calculation. The cycling condition and reaction components were as previously described [6]. The negative control consisted of nuclease-free water containing yeast RNA, and the positive control of SARS-CoV-2 RNAs used in a previous study [6,7]. The control RNA containing NIID-ORF1a set-targeting sequences (nucleotide position 595–1163 of EPI_ISL_408667) was prepared by same method [6,7]. The copy numbers of the control RNAs were calculated based on the molecular weight and the absorbance at 260 nm by using serial diluted RNA of more than 3 step and/or fluorescence using QuantiFluor RNA system and Quantus Fluorometer (Promega. Madison, WI, USA) (*n* = 3). The absorbance was measured by three times serially diluted RNA and the average was used (*n* = 3). The copy number adjusted control RNA was diluted with PCR-grade water containing RNase inhibitor (1 U/μL) and yeast RNA (10 ng/mL) as carrier for stable storage at −80 °C. Standard curves were generated by measuring serially diluted control RNAs. In the validation assays, the viral RNA copy number was calculated based on the standard curve obtained with the NIID-N2 set. In this study, the analytical sensitivity was defined as the 50% detectable copy number (per reaction) calculated by the Reed-Müench method (*n* = 4) and used the definition through the study. 

### 4.4. Spike Experiment

In the spike assay, virus stocks, which was centrifuged supernatant of infected cells, were treated with RNase A (Nippongene, Tokyo, Japan) at a concentration of 10 μg/mL for 60 min at 37 °C to exclude miscellaneous RNA other than viral RNA in the virion. The treated viral RNA was then extracted with TRIzol LS reagent, and the copy number was calculated by real-time RT-PCR as described above. The virus stocks were serially diluted based on the calculated copy number using virus-transport medium-containing nasopharyngeal swabs that showed negative for respiratory viruses. The dilutants were inoculated onto VeroE6 and VeroE6/TMPRSS2 cells in 96-well plates. After 90 min of adsorption, cells were washed with PBS and incubated with 1%FBS-DMEM at 37 °C in a 5% CO_2_ incubator. After 4 days of incubation, the cytopathic effect (CPE) of each well was recorded. 

### 4.5. Virus Isolation

Prior to the virus isolation, RNA was extracted from the specimens using the QIAamp viral RNA mini kit (Qiagen) and the copy number for each targeted region (ORF1a, S, and N) was determined by real-time RT-PCR using the control RNA templates as described above. Although the volume depended on the concentration and total volume of the specimen, specimens containing at least 100 copies, determined using the NIID-N2 primer/probe set, were inoculated onto VeroE6/TMPRSS2 cells in 24-well plates. Although the maximum of the specimen volume-inoculated depended on the RNA concentration and the amount, specimens containing 100, 200, 400, 800, and 1600 copies were inoculated in each specimen (1 serial dilution for a specimen). After 90 min of adsorption, the cells were washed and then incubated with 1% FBS-DMEM-containing antibiotics (penicillin-streptomycin, kanamycin, and fungizone) at 37 °C in a 5% CO_2_ incubator. During 4 days of incubation, wells with a CPE were considered as isolation positive. 

### 4.6. Evaluation of Primer/Probe Mismatches of NIID-Assays for Omicron Variants

Omicron variants of SARS-CoV-2 genomic sequences deposited from 1 to 13 February 2020 were collated from EpiCoV in the GISAID database (https://www.gisaid.org/ accessed on 27 February 2022). The data were collected checking “complete” and “low coverage excl” checkboxes and input “human” in the host field. Each sequence was aligned based on the Wuhan-Hu-1 isolate (GenBank: MN908947) using the multiple alignment program for amino acid or nucleotide sequences (MAFFT version 7, https://mafft.cbrc.jp/alignment/server/add_fragments.html?frommanual, accessed on 27 February) [38]. Mismatches in targeted region sequences were analyzed using SEQUENCHER software (Gene Codes, Ann Arbor, MI, USA) with these alignments. Sequences containing “N” in the targeted region were excluded from the analysis. To assess the effect of mismatches for the analytical sensitivity, the site-direct mutagenesis technique was used to generate mutated RNA template as previously described [7,26]. 

### 4.7. Statisitcal Analysis

Statistical analysis was performed using SigmaPlot version 14.5 (Systat Software Inc., San Jose, CA, USA). The *p* value < 0.05 was considered as significant.

## Figures and Tables

**Table 1:** Analytical sensitivity of NIID-ORF1a set.

Template		Analytical Sensitivity (Copy Number)		
		NIID-N2	NIID-S2	NIID-ORF1a
Synthesized control RNA		1.4	1.4	2.5
	RNase treatment			
Viral RNA (WK-521)	−	6.5	4.4	14.1
	+	2.5	4.4	4.4

**Table 2:** Analytical sensitivity of VeroE6/TMPRSS2 for SARS-CoV-2 isolation.

			Copy Number Required for Virus Isolation (Log Copies, *n* = 6)		
Virus	GISAID Number	Type	VeroE6	VeroE6/TMPRSS2	*p* Value
hCoV-19/Japan/TY-WK-521/2020	EPI_ISL_408667	Wuhan	2.8 ± 0.7	2.1 ± 0.6	0.0961
hCoV-19/Japan/QH-328-073/2020	unregistered	D614G	2.4 ± 0.7	1.6 ± 0.7	0.0302 *
hCoV-19/Japan/QHN001/2020	EPI_ISL_804007	Alpha	3.2 ± 0.0	1.9 ± 0.6	<0.0001 **
hCoV-19/Japan/TY8-612-P1/2021	EPI_ISL_1123289	Beta	3.2 ± 0.0	2.3 ± 0.5	<0.0001 **
hCoV-19/Japan/TY11-330-P1/2021	EPI_ISL_2158613	Kappa	3.5 ± 0.5	1.4 ± 1.2	0.0017 **
hCoV-19/Japan/TY11-927-P1/2021	EPI_ISL_2158617	Delta	1.9 ± 0.5	0.5 ± 0.5	0.0012 **
		average	2.8	1.6	0.0074 **

* *p* < 0.05; ** *p* < 0.01.

**Table 3:** Virus isolation from low copy number specimens.

		Cp Value			Log Copy Number/Reaction (11.7 µL of Specimen) *			
No	Specimen Type	NIID-N2	NIID-S2	NIID-ORF1a	NIID-N2	NIID-S2	NIID-ORF1a	Isolation
1	Pharyngeal swab	28.51	28.89	34.43	3.34	3.63	2.29	+
2	Pharyngeal swab	28.91	30.43	32.75	3.22	3.19	2.82	-
3	Pharyngeal swab	29.00	29.71	31.54	3.19	3.39	3.21	-
4	Pharyngeal swab	29.65	30.46	32.73	2.98	3.18	2.83	-
5	Pharyngeal swab	29.66	30.83	33.65	2.98	3.07	2.54	-
6	Pharyngeal swab	29.68	30.57	33.19	2.97	3.15	2.68	-
7	Pharyngeal swab	29.72	30.72	33.60	2.96	3.11	2.55	-
8	Pharyngeal swab	30.10	30.73	33.93	2.84	3.10	2.45	+
9	Pharyngeal swab	30.11	30.77	32.36	2.84	3.09	2.95	-
10	Pharyngeal swab	30.18	30.80	32.68	2.81	3.08	2.84	-
11	Nasal swab	30.29	32.15	34.60	2.78	2.70	2.23	+
12	Pharyngeal swab	30.39	31.06	32.89	2.75	3.01	2.78	-
13	Pharyngeal swab	30.42	31.07	32.91	2.74	3.01	2.77	-
14	Pharyngeal swab	30.51	30.70	33.26	2.71	3.11	2.66	-
15	Pharyngeal swab	30.51	32.16	35.19	2.71	2.69	2.04	-
16	Pharyngeal swab	30.61	32.00	34.45	2.68	2.74	2.28	-
17	Sputum	30.68	32.67	33.86	2.66	2.55	2.47	-
18	Pharyngeal swab	31.15	31.98	34.05	2.51	2.75	2.41	+
19	Pharyngeal swab	31.53	31.89	34.18	2.39	2.77	2.37	-
20	Pharyngeal swab	31.72	31.95	35.01	2.33	2.75	2.10	-
21	Pharyngeal swab	31.79	31.93	34.08	2.30	2.76	2.40	-
22	Pharyngeal swab	31.79	32.11	34.76	2.30	2.71	2.18	-
23	Pharyngeal swab	31.97	32.77	35.15	2.25	2.52	2.06	-
24	Pharyngeal swab	31.98	32.89	34.41	2.24	2.49	2.29	-
25	Pharyngeal swab	32.33	33.63	35.01	2.13	2.28	2.10	-
26	Pharyngeal swab	32.70	33.54	37.58	2.02	2.30	1.28	-
27	Pharyngeal swab	33.21	33.99	35.49	1.86	2.17	1.95	-
28	Pharyngeal swab	33.28	33.81	36.17	1.83	2.22	1.73	-
29	Nasal swab	33.36	34.23	36.51	1.81	2.10	1.62	-
30	Pharyngeal swab	33.57	34.35	36.69	1.74	2.07	1.57	-
31	Pharyngeal swab	33.96	33.47	34.86	1.62	2.32	2.15	-
32	Pharyngeal swab	33.99	33.71	36.94	1.61	2.25	1.49	-
33	Pharyngeal swab	34.03	36.04	-	1.60	1.59	-	-
34	Pharyngeal swab	34.15	34.25	35.93	1.56	2.10	1.81	-
35	Pharyngeal swab	34.22	35.07	37.86	1.54	1.86	1.19	-
36	Pharyngeal swab	34.23	36.29	37.68	1.53	1.52	1.25	-
37	Pharyngeal swab	34.33	34.29	39.17	1.50	2.09	0.78	-
38	Pharyngeal swab	34.50	33.54	35.47	1.45	2.30	1.96	-
39	Pharyngeal swab	34.72	35.05	37.09	1.38	1.87	1.44	-
40	Pharyngeal swab	34.77	34.93	37.36	1.36	1.90	1.35	-
41	Pharyngeal swab	34.83	35.44	37.80	1.34	1.76	1.21	+
42	Pharyngeal swab	35.46	36.15	-	1.14	1.56	-	-
43	Pharyngeal swab	35.64	36.15	-	1.09	1.56	-	-
44	Pharyngeal swab	35.86	36.14	-	1.02	1.56	-	-
45	Pharyngeal swab	35.97	36.43	-	0.98	1.48	-	-
46	Pharyngeal swab	36.19	38.53	-	0.91	0.88	-	-
47	Pharyngeal swab	36.26	35.80	37.88	0.89	1.66	1.19	-
48	Pharyngeal swab	36.26	-	-	0.89	-	-	-
49	Pharyngeal swab	36.83	38.05	37.06	0.71	1.01	1.45	+
50	Pharyngeal swab	36.97	-	-	0.67	-	-	-
51	Pharyngeal swab	37.00	-	-	0.66	-	-	-
52	Pharyngeal swab	37.09	-	37.35	0.63	-	1.36	-
53	Pharyngeal swab	37.25	37.62	-	0.58	1.14	-	-
54	Pharyngeal swab	38.67	-	-	0.13	-	-	-
55	Pharyngeal swab	-	35.90	38.30	-	1.63	1.05	-
56	Pharyngeal swab	-	34.86	-	-	1.92	-	-
57	Pharyngeal swab	-	37.66	-	-	1.12	-	-

* copy numbers were calculated by standard curve prepared by serially diluted each control RNA template.

## Supplementary Materials

The following supporting information can be downloaded at: https://www.mdpi.com/article/10.3390/pathogens11030302/s1, Table S1: Specific reaction of the NIID-ORF1a set, Table S2: Detection of SARS-CoV-2 by the NIID-ORF1a set in clinical specimens, Table S3: Primer/probe mismatches on NIID assays for Omicron variant.
