# Supplementary material for: Detection of the ORF1 Gene Is an Indicator of the Possible Isolation of Severe Acute Respiratory Syndrome Coronavirus 2
Source: Pathogens. 2022 Feb 27;11(3):302. doi: 10.3390/pathogens11030302 (PMC8953321; doi:10.3390/pathogens11030302)
Supplement: Supplementary file 1 [file pathogens-11-00302-s001.zip › pathogens-1589094-supplementary.pdf]

Table S1. Specific reaction of the NIID-ORF1a set.

Virus	Name of Isolate	GenBank Accession or GISAID ID	Amount	Results
SARS-CoV-2	Japan/TY/WK-521/2020	EPI_ISL_407084	2.5×10^{5} copies	+
SARS-CoV	Frankfurt-1	AY291315	5×10^{4} copies	-
MERS-CoV	EMC	NC_019843	2.5×10^{5} copies	-
HCoV-229E	VR-740	DQ243963	1.8×10^{9} copies	-
	Sendai-H/1121/04	AB691764	4.9×10^{6} copies	-
	Niigata/01/08	AB691767	2×10^{5} copies	-
	Fukushima/H829/2020	LC654445	2.1×10^{7} copies	-
	Fukushima/H832/2020	LC654446	6.8×10^{8} copies	-
HCoV-NL63	Amsterdam I	NC_005831	2×10^{9} copies	-
	Tokyo/SGH-15/2017	LC488390	2.7×10^{6} copies	-
	Tokyo/SGH-24/2018	LC488388	4.8×10^{5} copies	-
	Fukushima/H219/2018	LC654455	3.6×10^{7} copies	-
HCoV-OC43	VR-1558	AY391777	5.1×10^{10} copies	-
	Tokyo/SGH-36/2014	LC315646	6.8×10^{7} copies	-
	Tokyo/SGH-61/2014	LC315647	3.3×10^{8} copies	-
	Tokyo/SGH-06/2015	LC315648	1.1×10^{8} copies	-
	Tokyo/SGH-65/2016	LC315649	2.9×10^{8} copies	-
	Fukushima/H148/2018	LC654454	3.3×10^{7} copies	-
	Fukushima/H189/2018	LC654453	7.5×10^{8} copies	-
	Fukushima/H478/2019	LC654452	5.1×10^{9} copies	-
	Fukushima/H509/2019	LC654451	6.8×10^{8} copies	-
	Fukushima/O120/2017	LC654450	2.5×10^{9} copies	-
HCoV-HKU1	Tokyo/SGH-15/2014	LC315650	1.5×10^{8} copies	-
	Tokyo/SGH-18/2016	LC315651	3.3×10^{7} copies	-
	Fukushima/H815/2020	LC654447	3.0×10^{8} copies	-
	Fukushima/H821/2020	LC654448	1.5×10^{7} copies	-
	Fukushima/O943/2020	LC654449	7.0×10^{6} copies	-
ADV 3	G.B.	unregistered	7×10^{5} TCID50	-
ADV 4	RI-67	KX384949	7×10^{5} TCID50	-
ADV 7	Gomen	unregistered	7×10^{5} TCID50	-
Human respirovirus 1	PIV1, C-35	unregistered	4×10^{5} copies	-
	PIV1, NIID/79081/1/2019	LC654457	1.3×10^{3} copies	-
	PIV1, NIID/79082/2/2019	unregistered	2.4×10^{4} copies	-
Human respirovirus 3	PIV3, C-243	EU346887	5.1×10^{7} copies	-
	PIV3, NIID/79133/1/2019	LC654459	3.6×10^{3} copies	-
Human Rubulavirus 2	PIV2, NIID/56606/2/2019	LC654458	7.3×10^{2} $TCID_{50}$	-
	PIV2, NIID/56607/1/2019	unregistered	7.3×10^{1} $TCID_{50}$	-
Influenza viruses				
H1N1pdm	A/California/7/2009	unregistered	1×10^{5} copies	-
H3N2	A/Victoria/210/2009	unregistered	8.4×10^{4} copies	-
B	B/Brisbane/60/2008	unregistered	4.2×10^{5} copies	-
Human orthopneu-movirus	RSV, Long	AY911262	5.7×10^{4} copies	-
	RSV, A2	KT992094	2.5×10^{6} copies	-
	RSV, CH/18537	JX198143	4.8×10^{5} copies	-
	RSV, B1	AF013254	2.5×10^{3} copies	-

	RSV/A/NIID/2347/14	LC474556	5 $\times 10^4$ copies	-
	RSV/A/NIID/2367/14	LC474557	5 $\times 10^4$ copies	-
	RSV/A/NIID/2370/14	LC474558	5 $\times 10^4$ copies	-
	RSV/B/NIID/2472/14	LC474559	5 $\times 10^4$ copies	-
	RSV/B/NIID/2474/14	LC474560	5 $\times 10^4$ copies	-
Human metapneu-movirus	IA10-2003	DQ312451	7.5 $\times 10^6$ IU	-
	Sendai/0256/2015	unregistered	2 $\times 10^5$ IU	-
	Sendai/414/2013	unregistered	3 $\times 10^6$ FFU	-
	Sendai/1052/2011	unregistered	3 $\times 10^6$ IU	-

Table S2. Detection of SARS-CoV-2 by the NIID-ORF1a set in clinical specimens.

**Specimen No.**	**NIID-N2**	**NIID-S2**	**NIID-ORF1a**
1	-	-	-
2	-	-	-
3	-	-	-
4	-	-	-
5	-	-	-
6	-	-	-
7	-	-	-
8	-	-	-
9	-	-	-
10	-	-	-
11	-	-	-
12	-	-	-
13	-	-	-
14	-	-	-
15	-	-	-
16	23.3	24.15	26.44
17	25.08	25.87	28.58
18	27.4	28.15	30.7
19	29.68	30.57	33.19
20	29.65	30.46	32.73
21	28.91	30.43	32.75
22	30.51	32.16	35.19
23	32.70	33.54	37.58
24	35.86	36.14	-
25	36.19	38.53	-

Cp values were shown.

Table S3. Primer/probe mismatches on NIID assays for Omicron variant.

Primer/Probe Set		The Number of Available Sequences	The Number of Mismatched Sequences	Rate (%)	Distinctive Mismatch	The Number of Mismatched Sequences	Rate (%)
NIID-N2	Forward	347,853	527	0.152			
	Probe	345,046	411	0.119			
	Reverse	347,724	944	0.271			
		Total	1882	0.542			
NIID-S2	Forward	347,624	140	0.040			
	Probe	347,520	6262	1.802	T to G substitution on 11th nucleotide (TGCTCCTGCCATTTGTCATGATGG →TGCTCCTGCC**<u>G</u>**TTTGTCATGATGG)	5341	1.537
	Reverse	347,845	346	0.002			
		Total	6748	1.844			
NIID-ORF1a	Forward	287,892	34,845	12.103	T to G substitution on 18th nucleotide (GGAGCTGGTGGCCATAGTTA →GGAGCTGGTGGCCATAG**<u>G</u>**TA)	34,108	11.847
	Probe	287,925	469	0.163			
	Reverse	287,802	425	0.148			
		Total	35,739	12.414			
